# Supplementary material for: Functional Classification and Characterization of the Fungal Glycoside Hydrolase 28 Protein Family
Source: J Fungi (Basel). 2022 Feb 22;8(3):217. doi: 10.3390/jof8030217 (PMC8952511; doi:10.3390/jof8030217)
Supplement: Supplementary file 1 [file jof-08-00217-s001.zip › SF_merged.pdf]

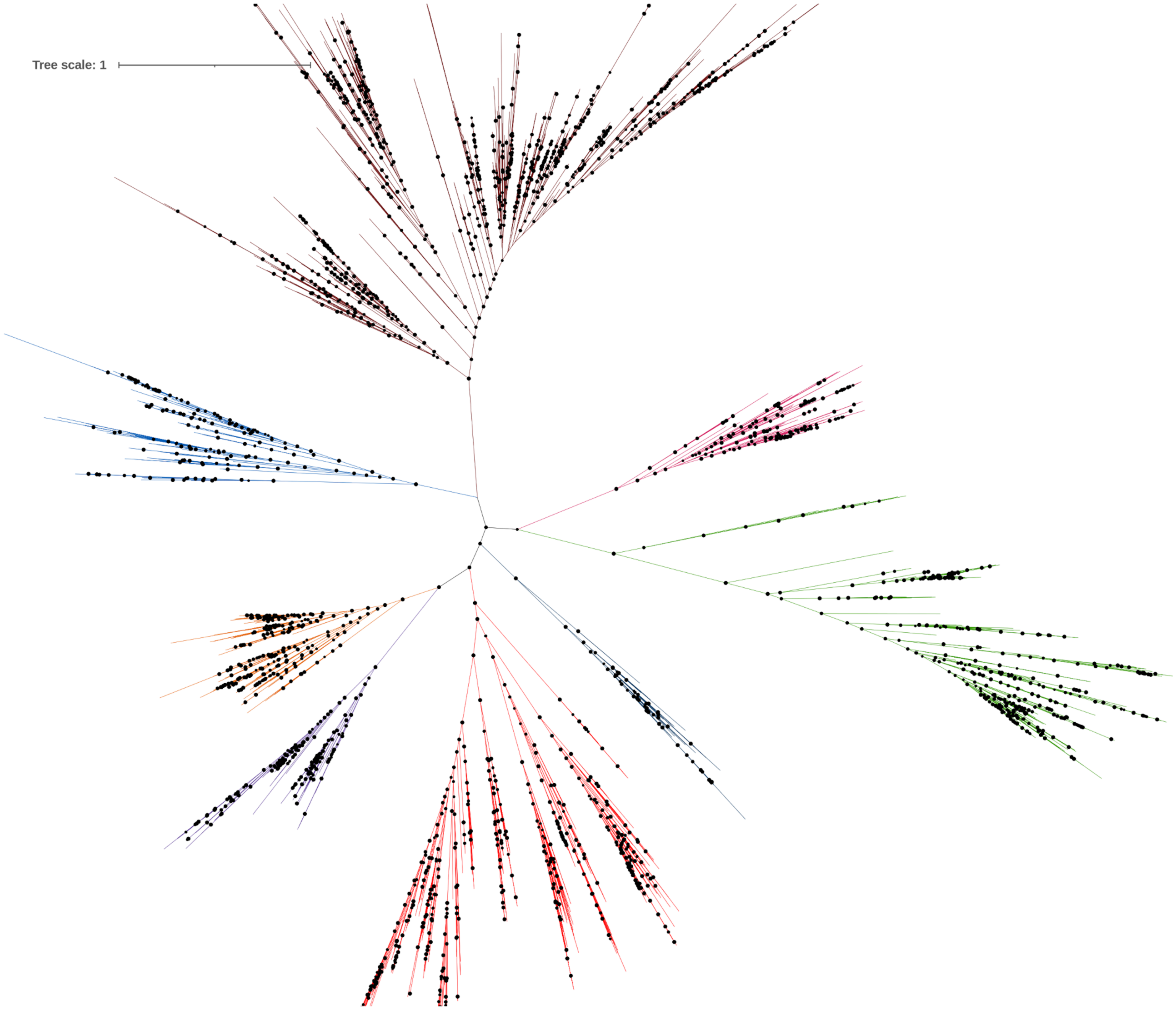

Supplemental figure S1: Bootstrap consensus tree of fungal GH28 homologues. The tree is the majority consensus tree of 1000 bootstrap trees. Dots shows normalized bootstrap support in between 0.8 and 1 (small to large). Colours of clades refer to subfamily classification as in Figure 2. The scale bar indicates 1 amino acid substitution per site.

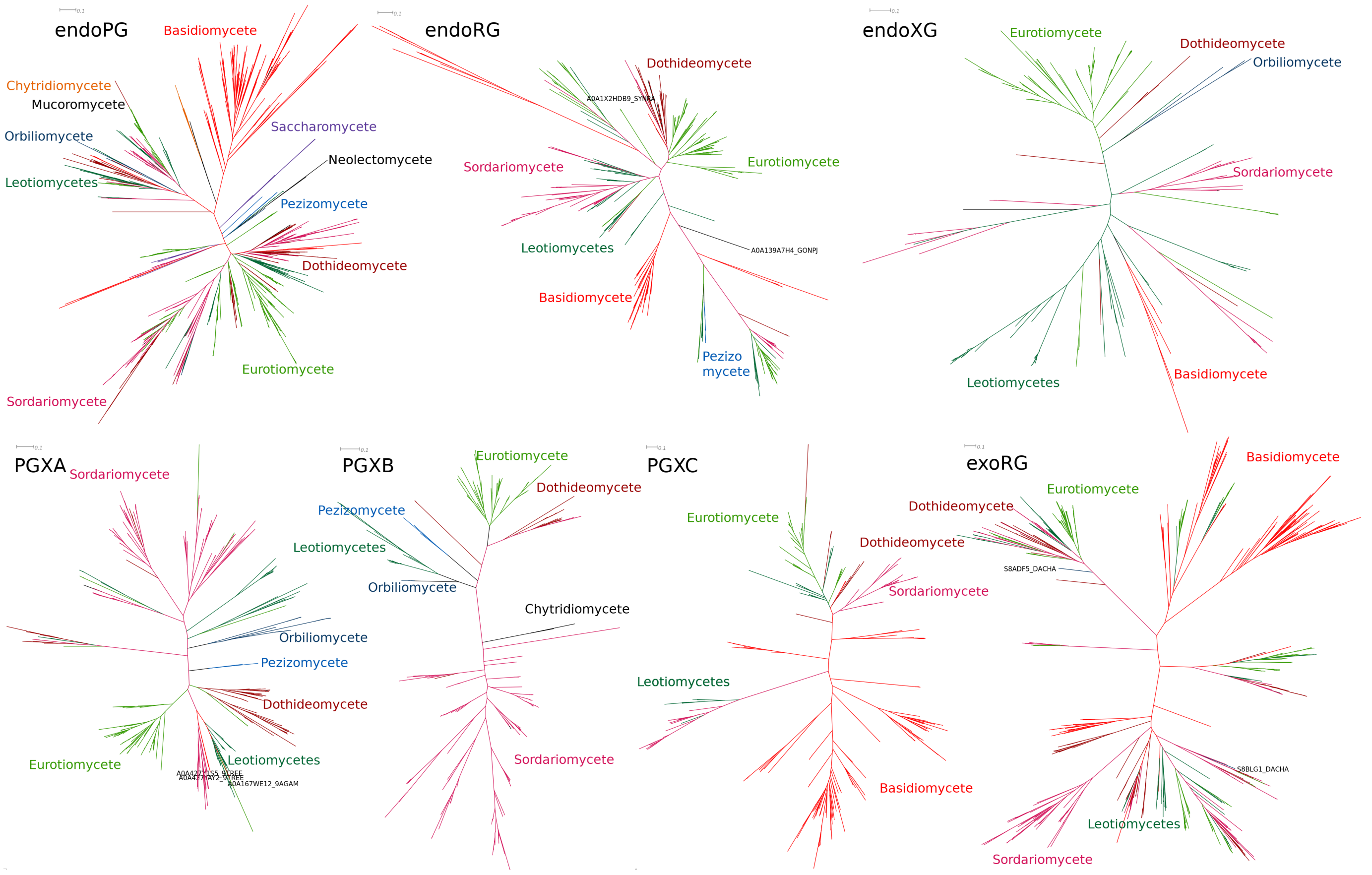

Supplemental figure S2: Taxonomic distribution of fungal GH28 subfamilies. Branch colours associate to taxa (ascomycete classes, basidiomycete), taxon names are in the same colour and placed nearby their major clade in each tree. The endoPG, endoRG and exoRG trees have been obtained by inception, the other trees are extracted from figure 2. EndoPG: The position of the major basidiomycete clade suggests two endoPG subfamilies exist; EndoRG: A0A139A7H4_GONPJ and A0A1X2HDB9_SYNRA are a mucoromycete and a chytridiomycete sequence respectively, pezizomycete sequences are only in a single clade; EndoXG: Orbiliomycete and pezizomycete sequences are only in a single clade; PGXA: Orbiliomycete and pezizomycete sequences are only in a single clade, A0A167WE12_9AGAM, A0A427YAY2_9TREE andA0A427Y1S5_9TREE are three basidiomycete sequences; PGXB: Chytridiomycete andorbiliomycete sequences are only in a single clade; exoRG: S8ADF5_DACHA andS8BLG1_DACHA are orbiliomycete sequences which, combined with the overall taxonomic distribution and topology, suggests this large subfamily has also evolved at least two distinct functional subfamilies.

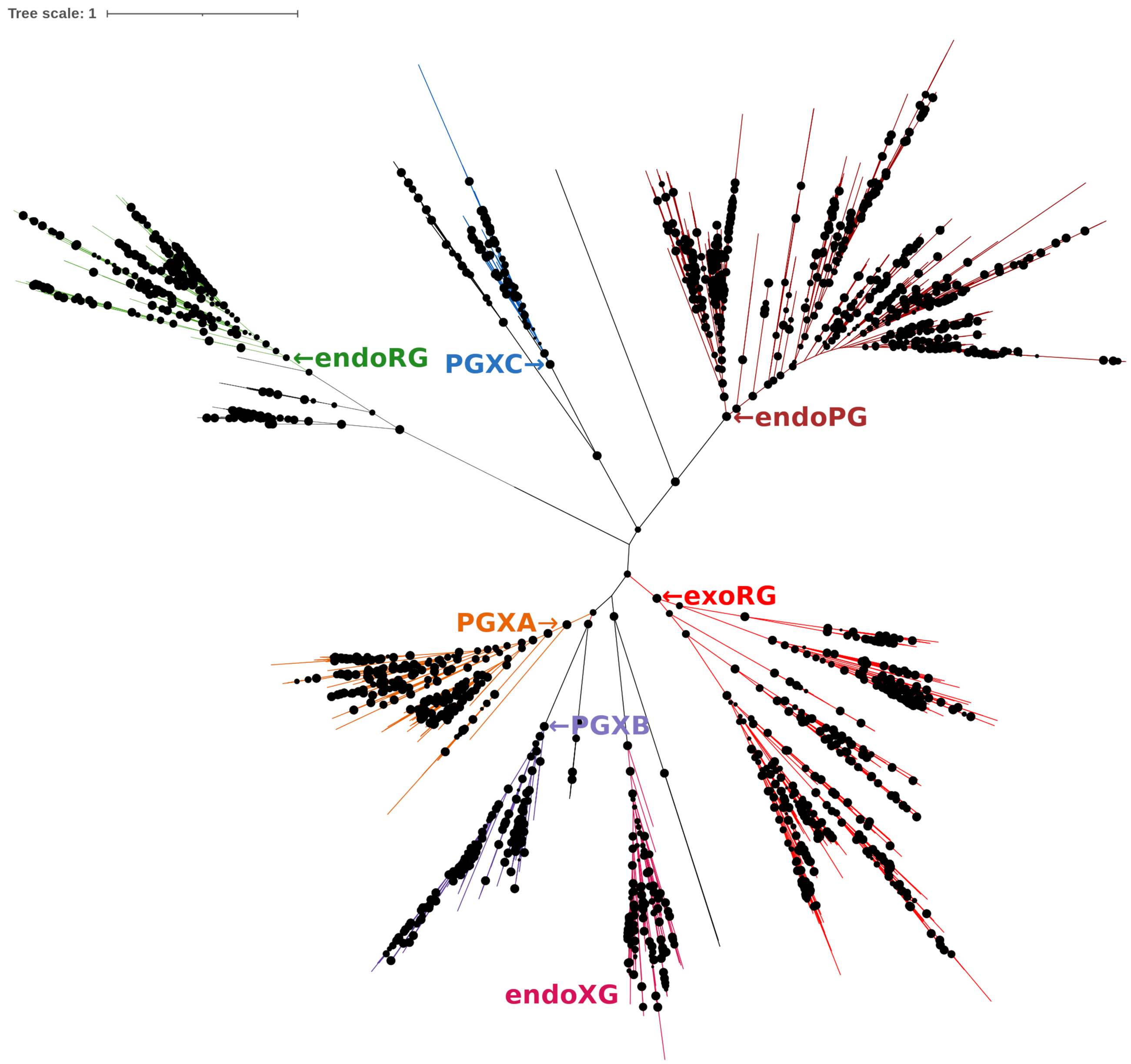

Supplemental figure S3: Bootstrap consensus tree of ascomycete GH28 homologues. The tree is the majority consensus tree of 1000 bootstrap trees. Dots shows normalized bootstrap support in between 0.8 and 1 (small to large). Colours of clades refer to subfamily classification as in Figure 2 and correspond to final datasets used for computational analyses. The scale bar indicates 1 amino acid substitution per site.

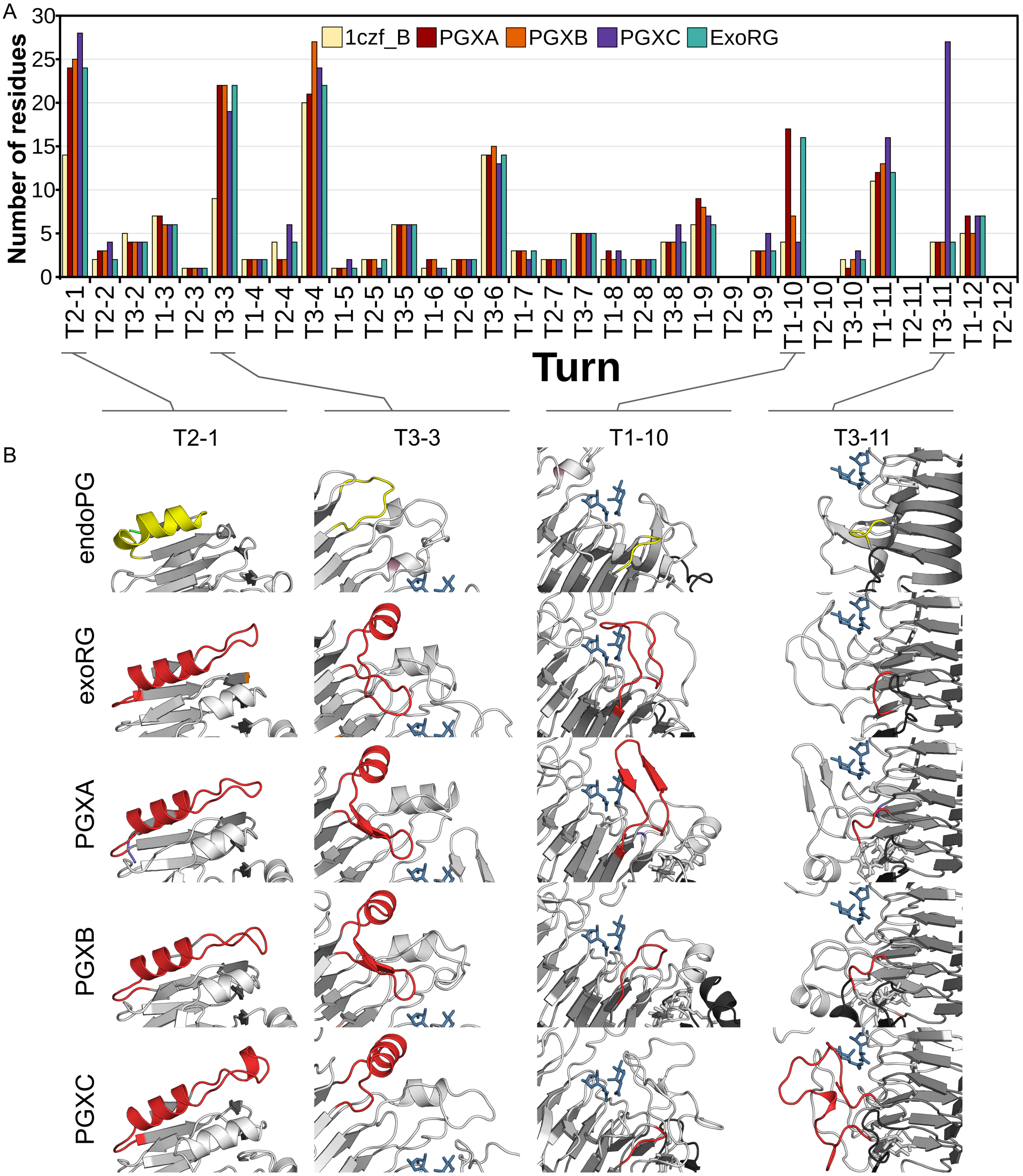

Supplemental figure S4: Loop regions of exomode enzymes. **A:** Number of amino acids in turns for exomode reference sequences compared to the homologous turns in endoPG reference 1CZF. **B:** Turns indicated in **A** (columns) highlighted in reference structures for endoPG and exomode subfamilies (rows).

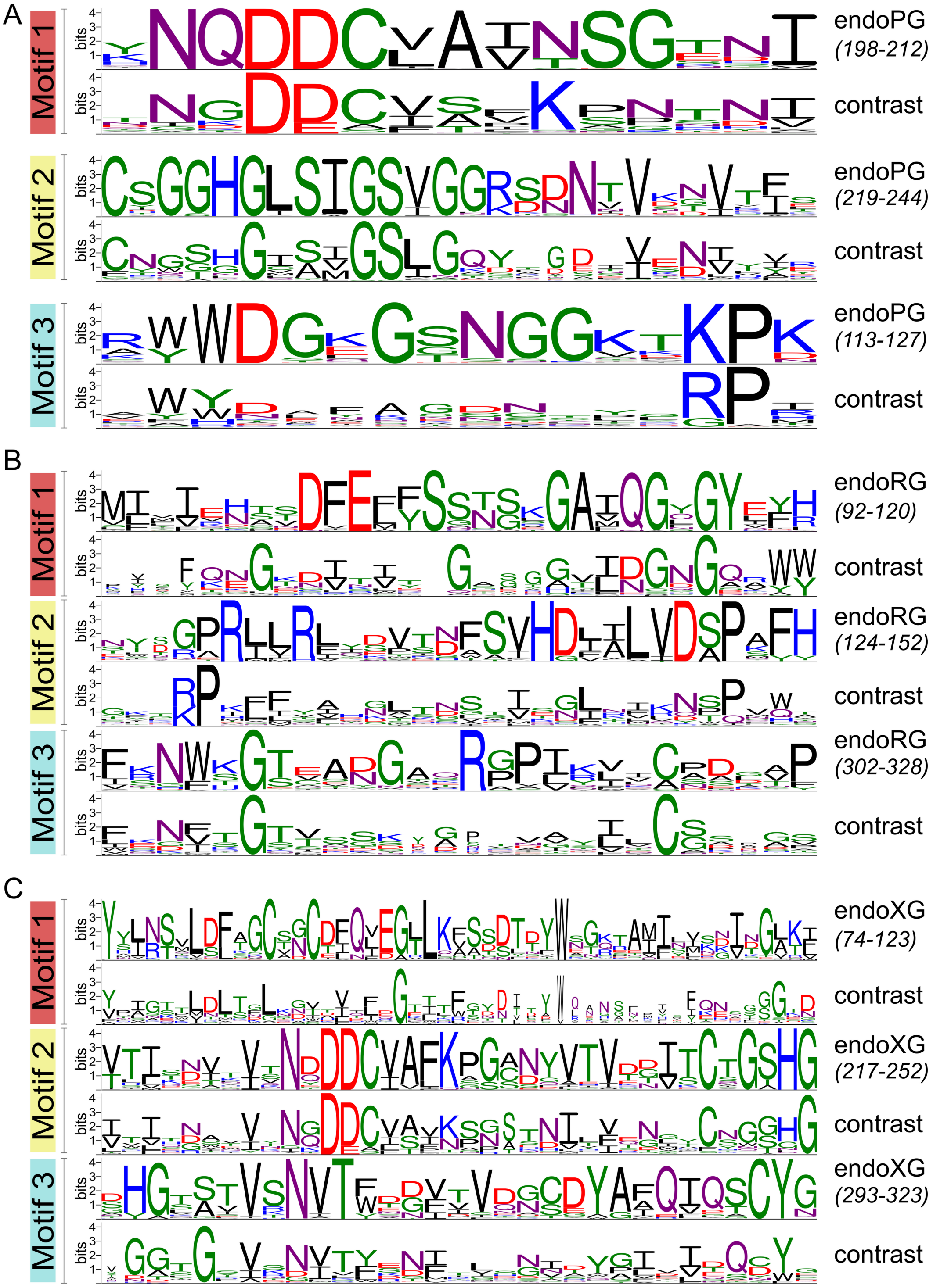

Supplemental figure S5: Logos for endoPG (**A**), endo RG (**B**), endoXG (**C**), exoRG (**D**), PGXA (**E**), PGXB (**F**) and PGXC (**G**) classes. Shown are the logos for the top 3 motifs as described in the main text. Motif numbering (in parentheses) corresponds to position in the reference sequence for each class. Reference sequences: endoPG 1CZF; endoRG 1RMG; endoXG 4C2L; exoRG A0A194WYS5_9HELO; PGXA PGLRX_ASPFU; PGXB Q7SAI8_NEUCR; and PGXC A0A1L9N956_ASPTC. Panels **D** to **G** in next page.

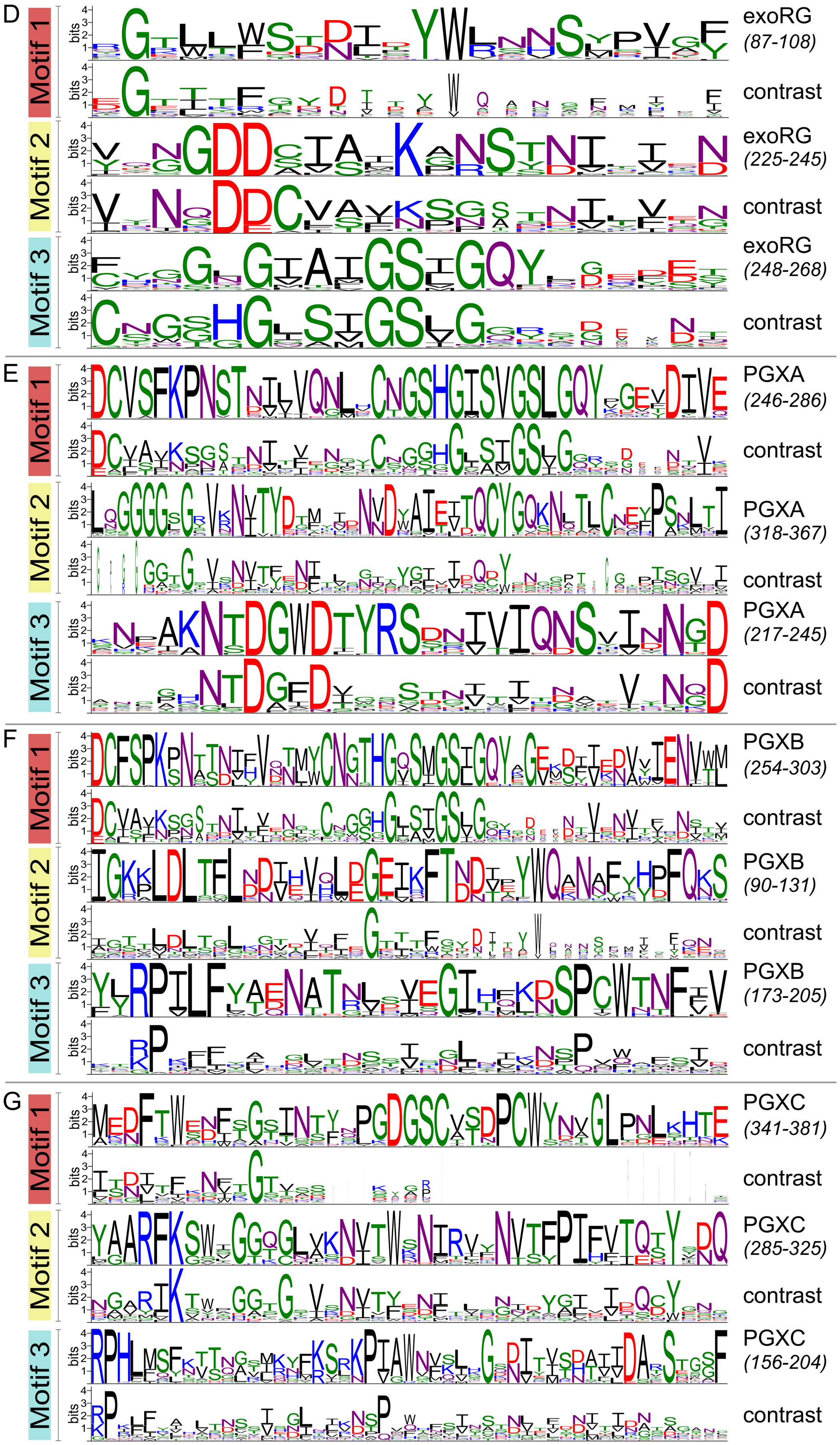

Supplemental figure S5 (continued).

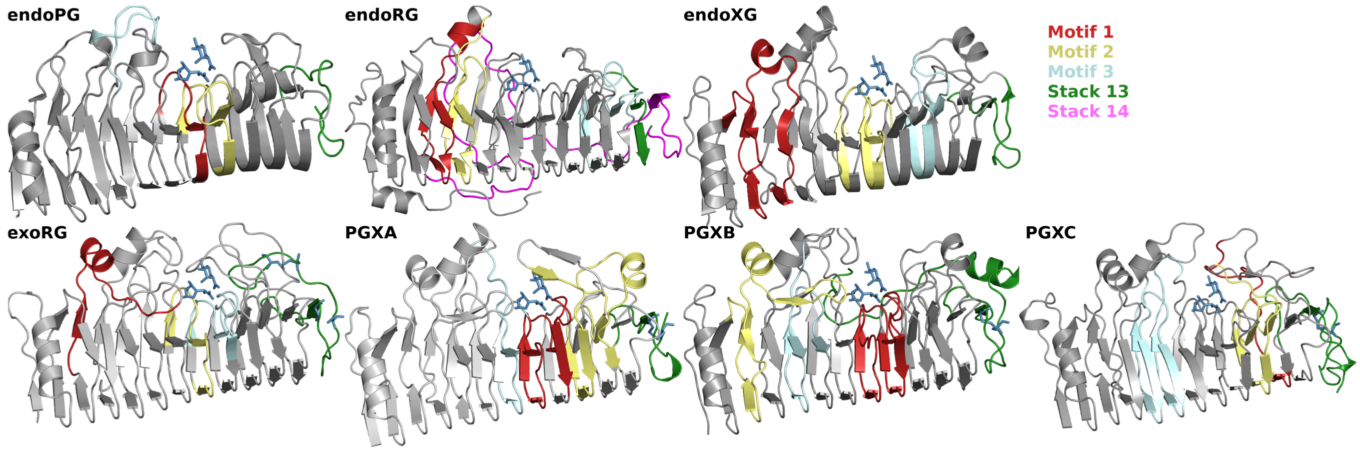

Supplemental figure S6: MEME motif locations for all subfamilies. Motif numbering corresponds to MEME output where the first motif is most significant. Motif regions and C-terminal differences are plotted on structure models for reference sequences: endoPG: 1CZF, endoRG: 1RMG, endoXG: 4C2L, PGXA: PGLRX_ASPFU, PGXB Q7SAI8_NEUCR, PGXC A0A1L9N956_ASPTC and exoRG: A0A194WYS5_9HELO.

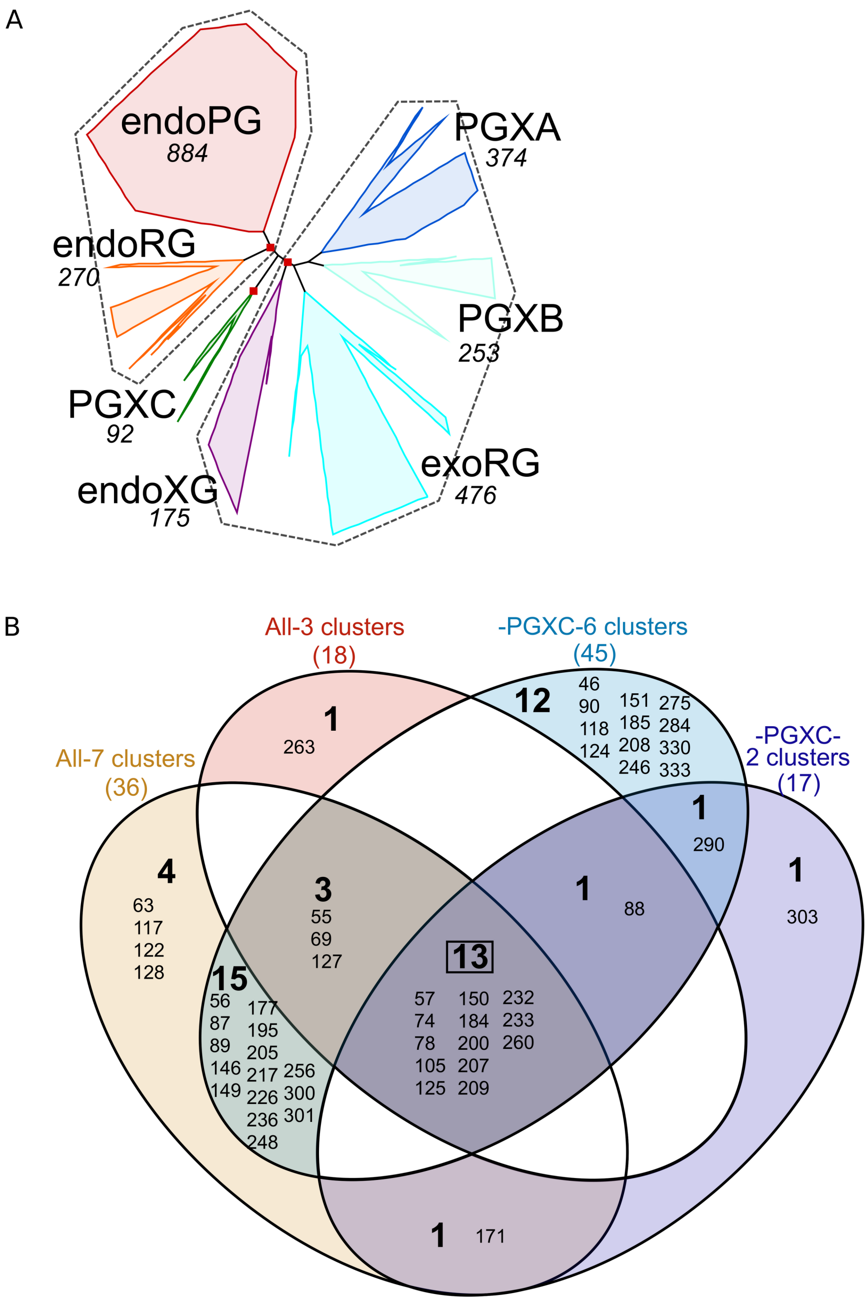

Supplemental figure S7: Global SDP analysis. **A:** The seven main functional classes are indicated in the ascomycete phylogeny (amount of sequences in each class is indicated). The MSA containing sequences from all classes was clustered into the different functional classes (All-7 clusters), or in three clusters based on the phylogeny (with the common ancestor indicated with a red square, All-3 clusters). In addition, we prepared the datasets excluding PGXC, resulting in six functional clusters (-PGXC-6 clusters) or two phylogenetic clusters (-PGXC-2 clusters, dashed lines). Based on this, we search for CDPs with SDPfox, calculated MI with MISTIC and determined the corresponding SDNs. **B:** Venn diagram with SDPs identified in each analysis described in **A**. Positions correspond to the endoPG reference 1CZF.

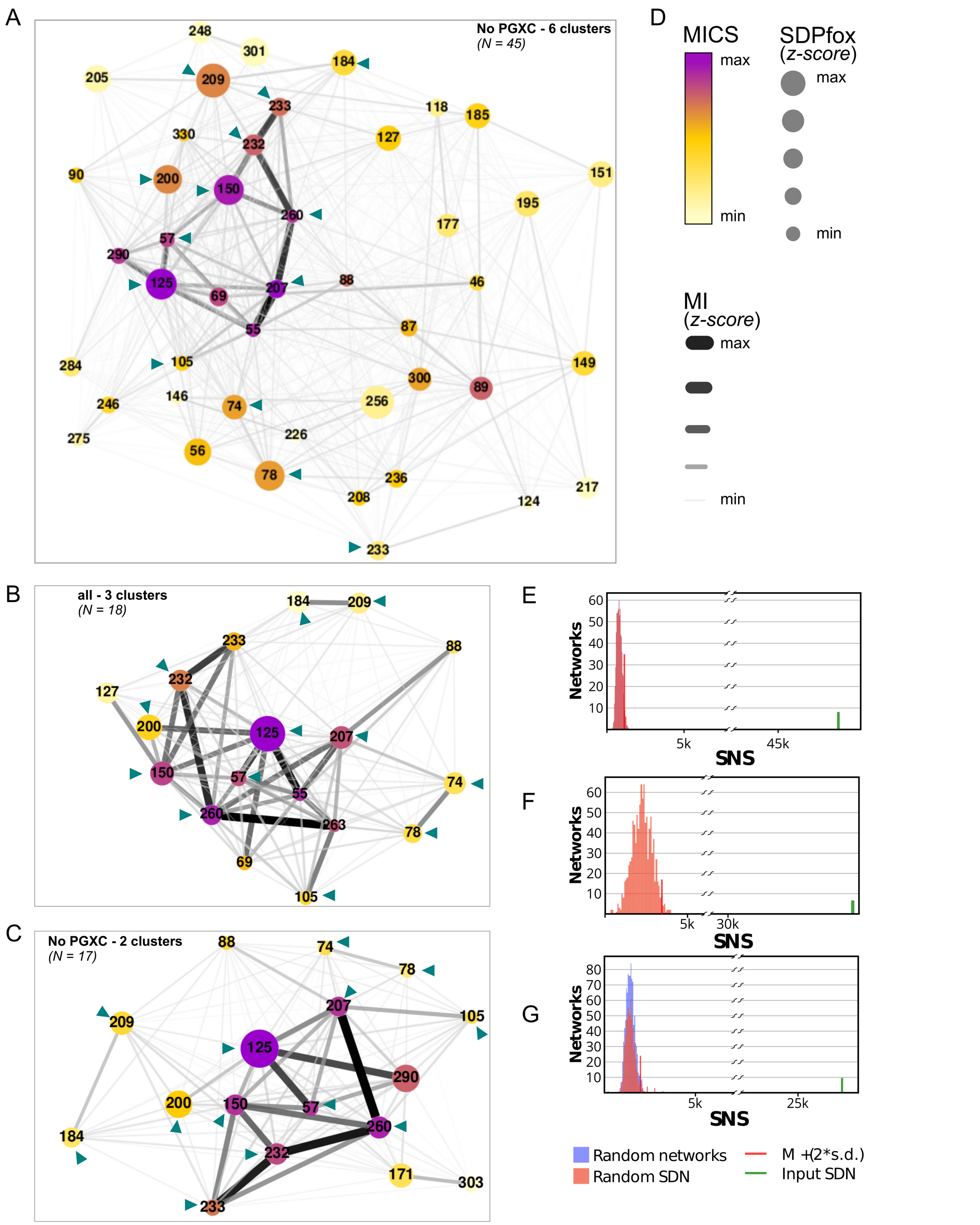

Supplemental figure S8: Additional major SDN analyses. SDN resulting from analyses with **A:** "6 clusters-No PGXC"; **B:** "All-3 clusters"; and **C:** "2 clusters-No PGXC". Positions denoted with a triangle indicate SDPs identified in all analyses. N indicates the number of SDPs or nodes. **D:** Scales used for node colour (indicating MICS) and diameter (SDPfox z-score), and edges thickness and colour (denoting MI z-score). **E, F, G:** Score distributions of 1000 random networks contrasted with the SNS of the SDNs in **A**, **B** and **C**, respectively. The respective z-scores of the SDNs are 350.3, 86.5 and 96.2. The "All-7 clusters" results are in Figure 6. Positions correspond to the endoPG reference 1CZF.

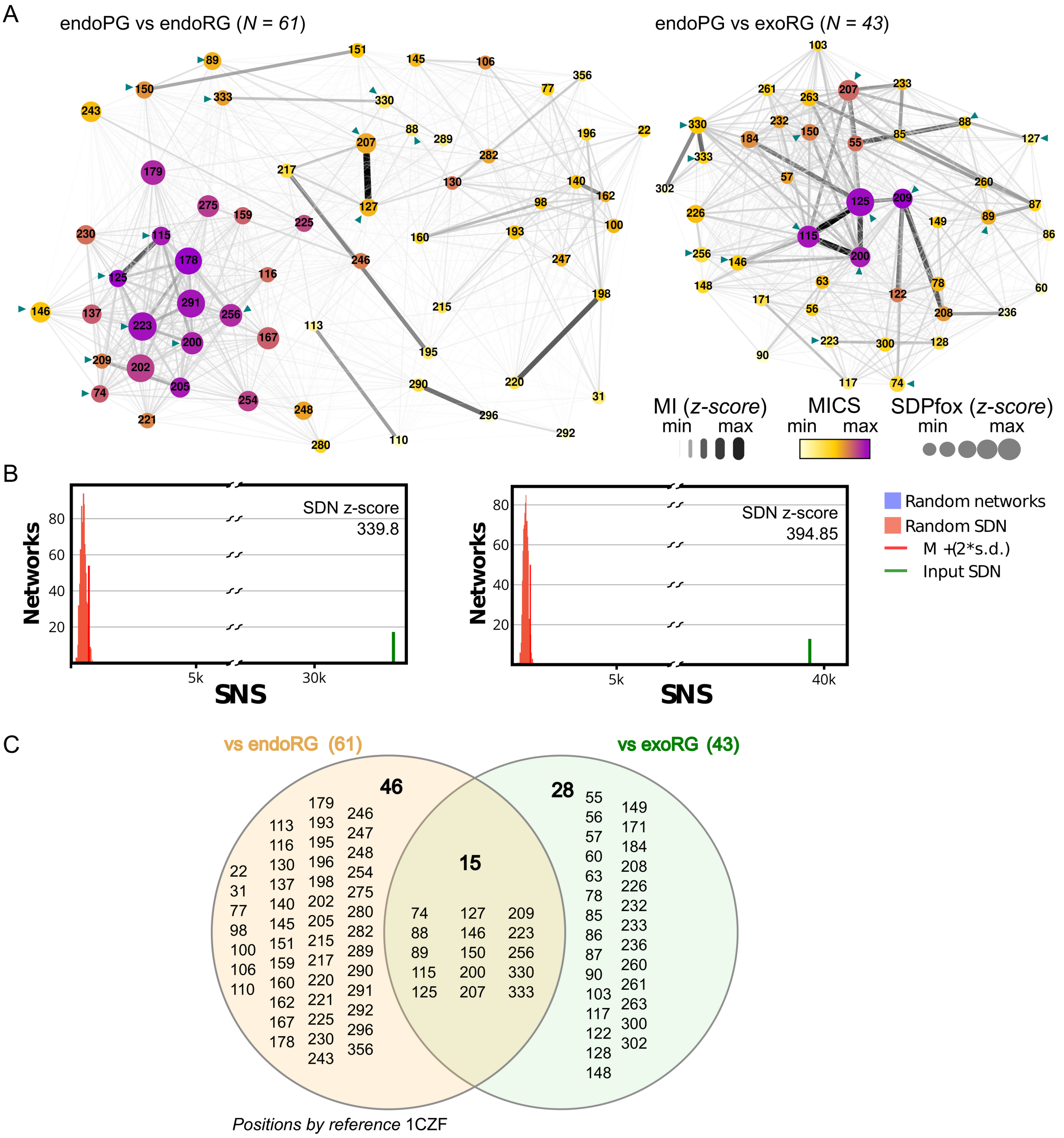

Supplemental figure S9: SDP identification for endoRG. **A:** SDN resulting from the analysis between endoPG vs endoRG (left) and endoPG vs exoRG (right). N indicates the node number in the SDN. Scales used for node colour (indicating MICS) and diameter (SDPfox z-score), and edges thickness and colour (denoting MI z-score) are shown. **B:** Score distributions of 1000 random networks contrasted with the SNS of the SDNs endoPG vs endoRG (left) and endoPG vs exoRG (right). **C:** Venn diagram of identified SDPs of endoPG vs either endoRG or exoRG. Positions correspond to the endoPG reference 1CZF.

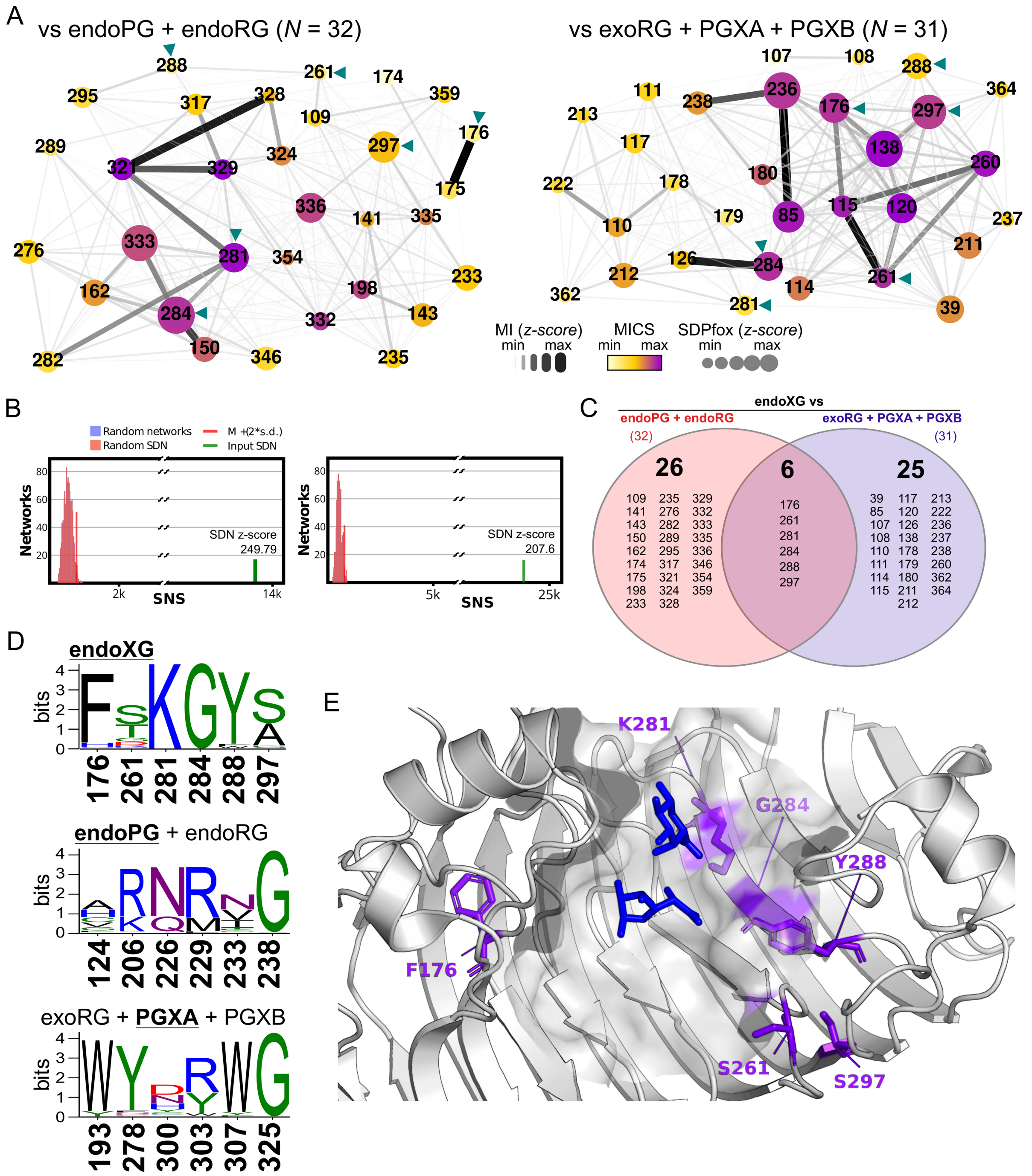

Supplemental figure S10: SDP analysis for endoXG. **A:** SDN resulting from the analysis between endoXG vs endoPG + endoRG (left) and endoXG vs exoRG + PGXA + PGXB (right). Scales used for node colour (indicating MICS) and diameter (SDPfox z-score), and edges thickness and colour (denoting MI z-score) are shown. Positions correspond to endoXG reference sequence 4C2L. Triangles indicate SDPs identified in both analyses. **B:** Score distributions of 1000 random networks contrasted with the SNS of the SDNs endoXG vs endoPG +endoRG (left) and endoXG vs exoRG + PGXA + PGXB (right). **C:** Venn diagram showing the SDPs identified in each analysis. **D:** Shared SDP logos. Positions correspond to the reference sequence of each class (underlined for each contrast). **E:** Mapping of identified SDPs in both analyses on endoXG reference structure 4C2L aligned to 1KCD, galacturonate ligands in blue sticks only. Surface region corresponding to the binding cavity is also shown. Panels **F** to **I** in next page. **F, G:** Logos of unique SDPs from endoXG vs endoPG + endoRG and endoXG vs exoRG +PGXA + PGXB analyses, contrasting the reference sequences in each subset (underlined). Circles indicate SDPs with high conservation in endoXG. **H:** Mapping of unique SDPs: endoXG vs endoPG + endoRG (endomode, red); and endoXG vs exoRG + PGXA + PGXB (exomode, cyan). **I:** Mapping of unique SDPs from endoXG vs exoRG + PGXA + PGXB (exomode) sourrounding PB1 (in yellow, PB1-9 in orange). Non-SDP C88 shown in black. Shown are endoXG reference structure 4C2L aligned to1KCD, galacturonate ligands in blue sticks only.

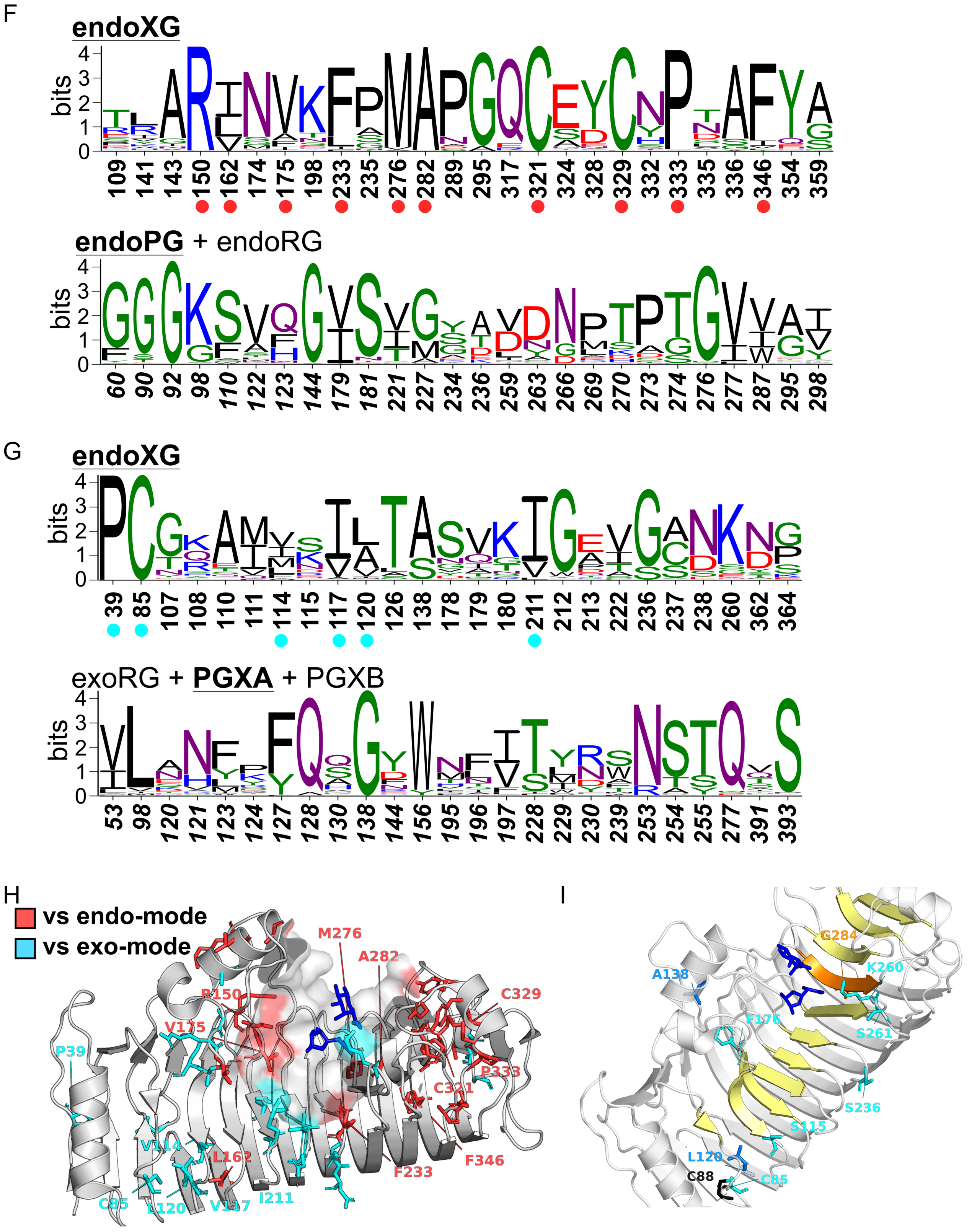

Supplemental figure S10 (continued).

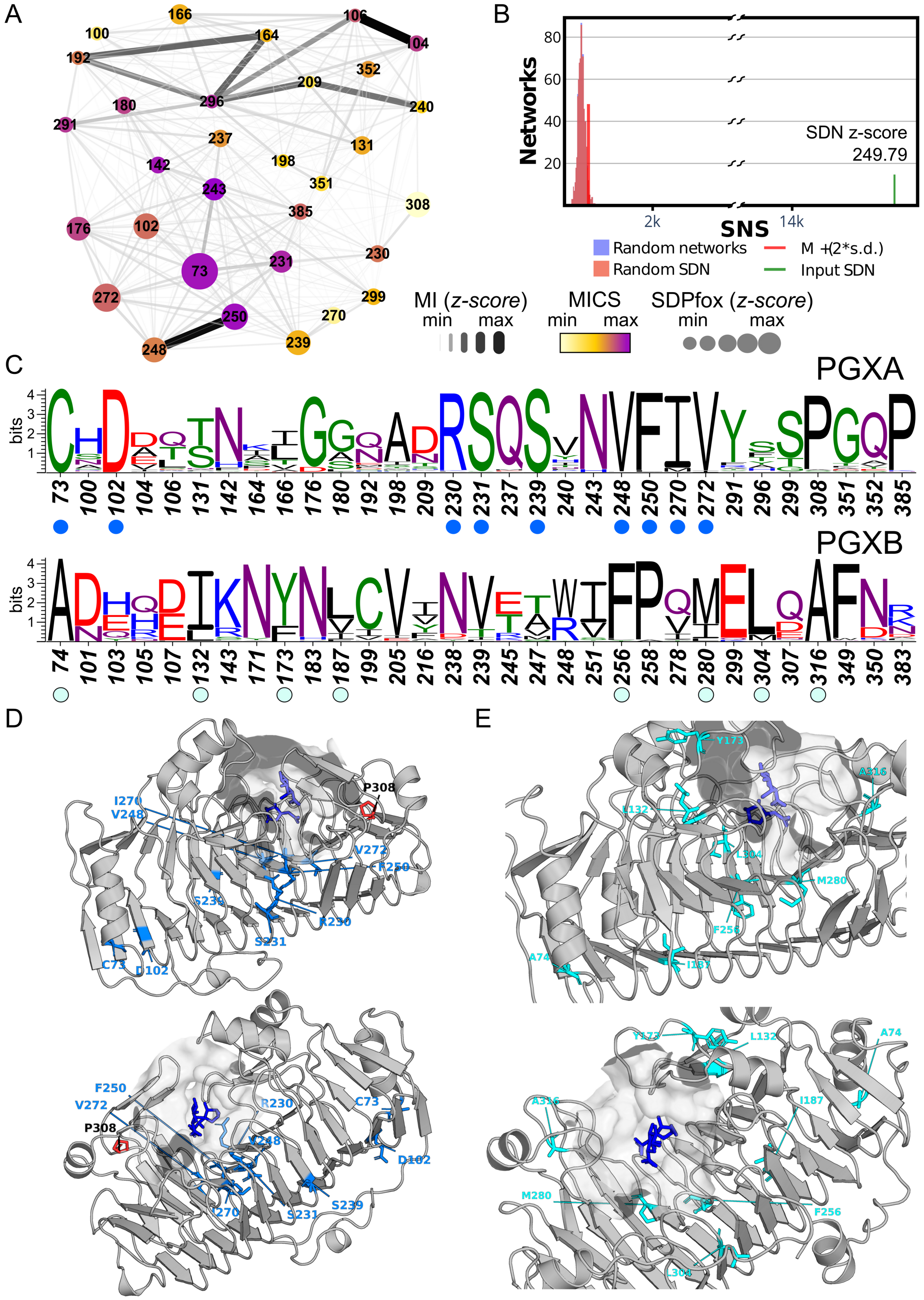

Supplemental figure S11: SDP analysis for PGXA vs PGXB. **A:** SDN resulting from the analysis between PGXA vs PGXB. Scales used for node colour (indicating MICS) and diameter (SDPfox z-score), and edges thickness and colour (denoting MI z-score) are shown. Positions correspond to PGXA reference sequence PGLRX_ASPFU. **B:** Score distributions of 1000 random networks contrasted with the SNS of the SDN PGXA vs PGXB. **C:** Logos of SDPs identified in PGXA vs PGXB analysis. Positions correspond to the reference sequence of each class. Coloured circles denote residues with conserved physicochemical properties at a given position. **D, E:** mapping of identified SDPs in reference sequences for PGXA (**D**) and PGXB (**E**). Two rotational views are shown. Residues with high conservation are labelled. P308 is shown in PGXA. Structures are aligned to 1KCD (galacturonate substrates in blue sticks only). Surface region corresponding to the binding cavity is also shown.

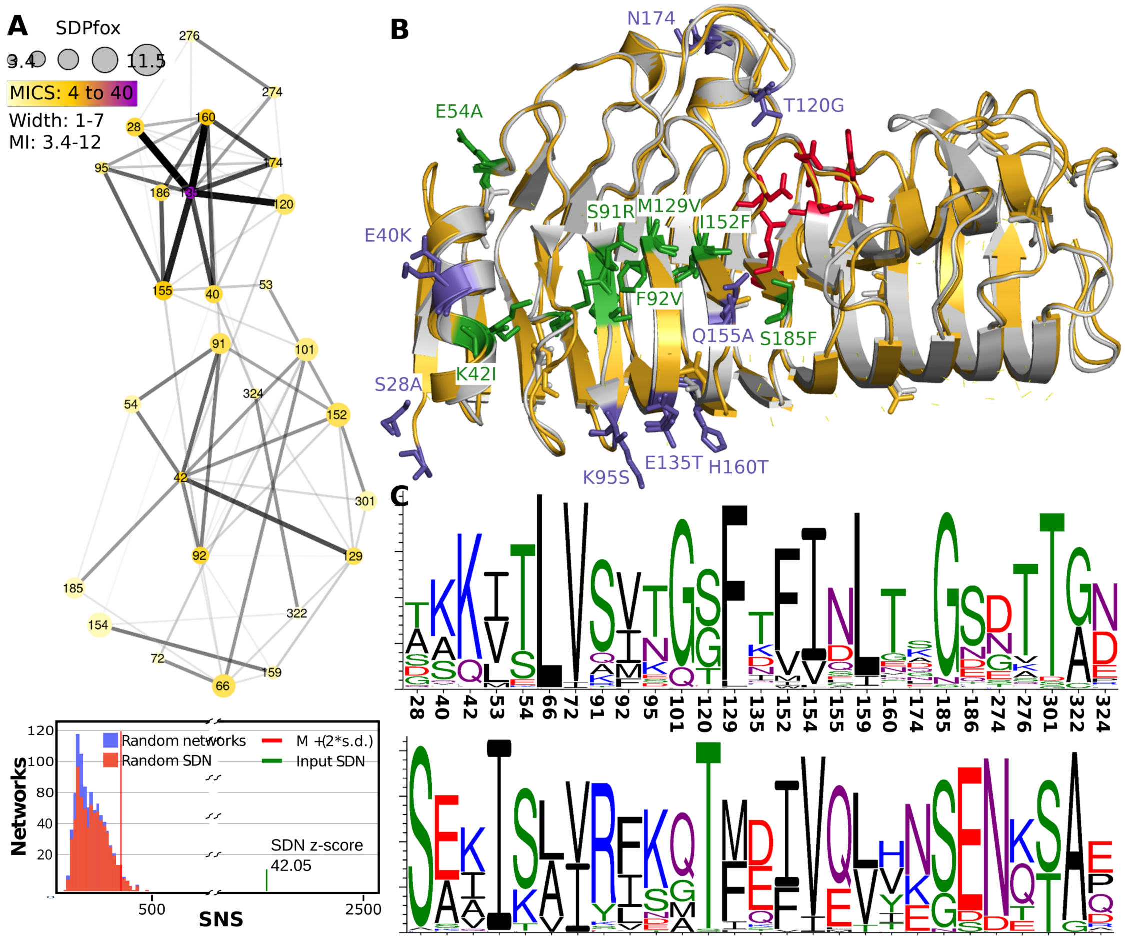

Supplemental figure S12: Processive endoPGA differs from Non-Processive endoPGA around subsites -5 to -1. **A:** SDN resulting from the analysis between processive endoPGA vs non-processive endoPGA. The graph shows score distributions of 1000 random networks contrasted with the SNS of the processive endoPGA vs non-processive endoPGA. **B:** Structure analysis. Shown are structures of 1NHC (processive, grey cartoon) and 1IA5 (non-processive, gold cartoon). The major SDPs centered around SDP135 as well as SDP91 are shown in purple and green stick, respectively (1NHC, numbering according to 1IA5). Catalytic residues D181, D183, D201, D202 and H223 are shown in red stick. **C:** SDP logos of the processive (top) and non-processive (bottom) clusters. For clustering see Figure 8. All numbers according to 1CZF.

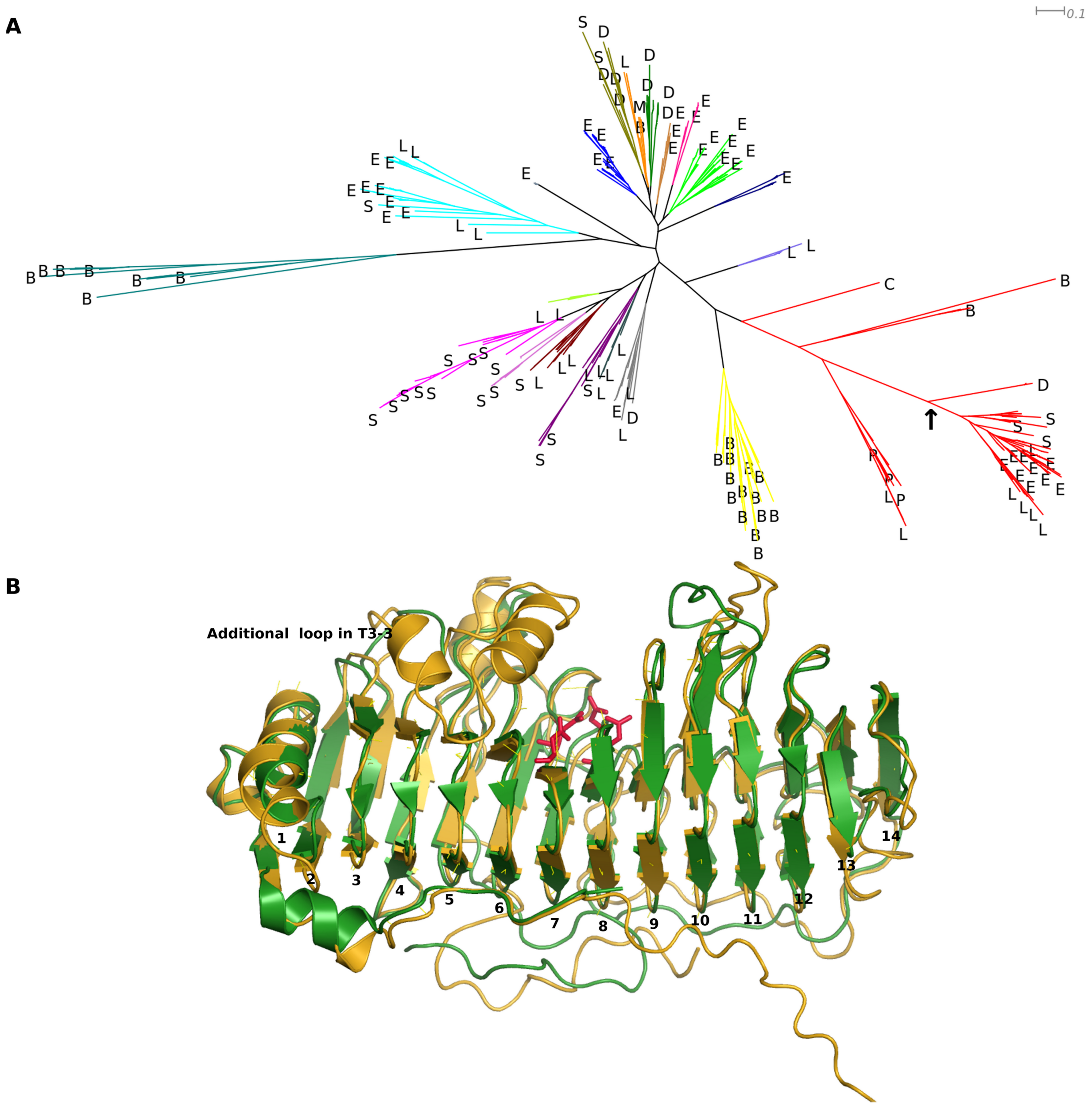

Supplemental figure S13: A novel GH28 subfamily that has evolved from the endoRG subfamily. **A:** HMMERCTTER clustering of the endoRG subfamily. Most HMMERCTTER clusters are taxonomically biased. B: basidiomycete; C: chytridiomycete; D: dothideomycete, E: eurotiomycete; L: leotiomycete; M: mucoromycete; P: pezizomycete; S: sordariomycete, Y: saccharomycete. The arrow indicates the common ancestor of a subfamily with an additional subsequence in T3-3. **B:** Structural alignment of the best Alphafold model of sequence A0A0S7E1Z2_9EURO (gold cartoon) and the endoRG from *A. aculeatus* PDB identifier 1RMG (green cartoon). The catalytic Asp177, Asp180, Asp197 and Glu198 are in red stick. Numbers below the structure alignment indicate stacks.
